# Empowering ECRs to make research projects flourish: lessons from a European research project

**DOI:** 10.12688/openreseurope.21517.1

**Published:** 2025-10-15

**Authors:** Julius Schlumberger, Kelley De Polt, Judith N. Claassen, Timothy Tiggeloven, Sophie L. Buijs, Marleen C. de Ruiter, Maria Vittoria Gargiulo, Joel C. Gill, Núria Pantaleoni Reluy, Robert Šakić Trogrlić, Philip J. Ward

**Affiliations:** 1 Climate Adaptation and Disaster Risk Department, Deltares, Delft, South Holland, 2629 HV, The Netherlands; 2 Department of Water & Climate Risk, VU University Amsterdam Institute for Environmental Studies, Amsterdam, North Holland, 1081 HV, The Netherlands; 3 Department of Biogeochemical Integration, Max Planck Institute for Biogeochemistry, Jena, Thuringia, 07745, Germany; 4 Dipartimento di Fisica “E.R. Caianiello”, Universita degli Studi di Salerno, Fisciano, Campania, 84084, Italy; 5 Cardiff University School of Earth and Environmental Sciences, Cardiff, Wales, CF10 3AT, UK; 6 Department of Civil and Environmental Engineering, Universitat Politècnica de Catalunya, Barcelona, Catalonia, 08034, Spain; 7 International Institute for Applied Systems Analysis, Laxenburg, Lower Austria, A-2361, Austria

**Keywords:** Early Career Researchers, ECR, career development, project management; European research projects, mentorship, empowerment, research culture

## Abstract

Early Career Researchers (ECRs) are essential contributors to scientific innovation and research outcomes, yet their empowerment and development within research projects remains an underexplored area. While European and national initiatives provide valuable funding and career development opportunities, less attention has been given to how similar opportunities can be meaningfully integrated and supported within the structure of research projects themselves. Drawing on experiences from the EU Horizon 2020 project MYRIAD-EU, this perspective explores practical approaches to integrating ECR empowerment into collaborative, interdisciplinary research. Approximately 30% of the project consortium was made up of ECRs, whose involvement was facilitated through structural mechanisms such as the establishment of an ECR Board, direct representation in project management, and leadership opportunities within work packages. Additionally, ECRs co-organized dedicated events and actively fostered professional networks both within and beyond the project. These opportunities and activities benefitted the ECRs in multiple ways, including skill development and professional network formation. ECRs also offered various benefits to the project, including additional resources and ideas to successfully manage and conduct the project as a whole, as well as external recognition for its empowerment efforts. We showcase three types of activities, including structural involvement in project management, organizing events for ECRs, and efforts to form ECR networks. We identify and discuss three enabling factors that play a critical role in creating an empowering environment: advisory support, ECR agency, and other factors, such as project design. Within this perspective, we aim to encourage research projects and funding institutions to further build on these practices, ranging from low-hanging fruit to more high-effort, high-reward options, in order to foster environments where ECRs can grow into independent researchers with benefits for the projects as well.

## 1. Introduction

Early Career Researchers (ECRs) play a crucial role in the research process, often serving as the driving forces behind the implementation of a project’s research. Compared to their more senior colleagues, ECRs typically represent more diverse cohorts, bringing fresh perspectives, unchallenged idealism, and creativity that can drive the development of innovative scientific methods and ideas ( Campbell *et al*., 2019; Wilder *et al*., 2013). Additionally, ECRs often form the majority of the scientific workforce in research projects, making significant contributions to their outcomes and broader scientific impact ( Kent *et al*., 2022).

**Figure 1.**
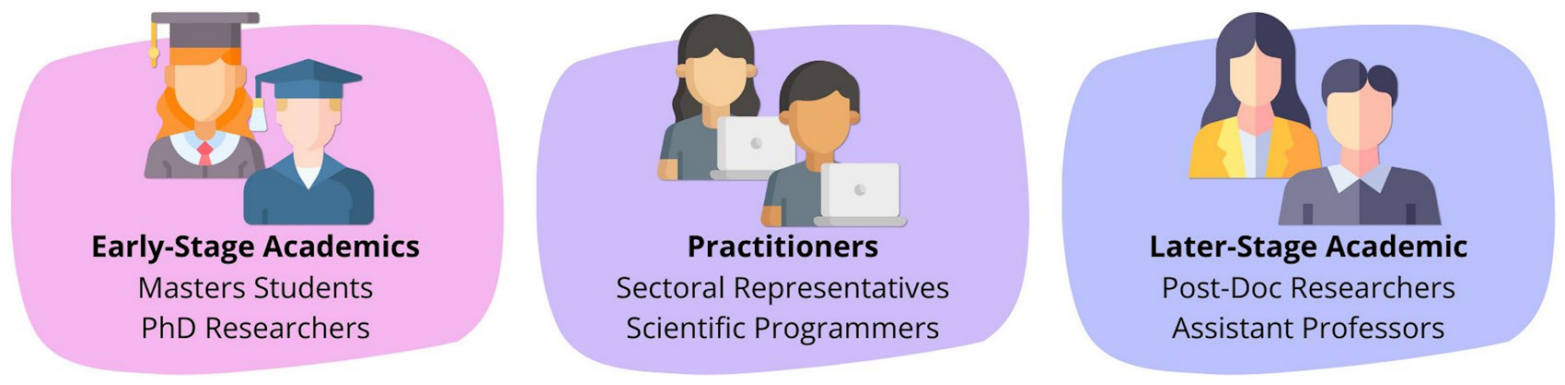
Profiles of ECRs include “Early-stage academics" (e.g., Master's students, PhD researchers), “Practitioners” (e.g., sectoral representatives, scientific programmers), and “Later-stage academics” (e.g., post-docs, assistant professors). *Icons by Freepik from Flaticon.com*.

The early career stage is a critical period of transition from a dependent to an independent researcher, during which ECRs develop and expand their skill set ( Laudel & Gläser, 2008; Nicholas *et al*., 2019). ECRs can be defined in different ways, but in this perspective, we use the European Geosciences Union definition, which defines ECRs as students, PhD candidates, and practicing researchers who obtained their highest degree (e.g., BSc, MSc, or PhD) within the past seven years (EGU, 2025; https://www.egu.eu/awards-medals/ecs-definition/). Figure 1 illustrates some examples of who is considered an ECR according to this definition. Browning *et al*. (2017) identify key factors that support successful career development for ECRs, including formal and informal mentoring, access to research funding, participation in active research groups, opportunities to teach or supervise students, and formal leadership training. Beyond individual skill development, Kent *et al*. (2022) emphasize the importance of an empowering research culture - one in which ECRs feel they have the authority and opportunity to contribute meaningfully. This requires not only personal agency but also supportive supervisors, colleagues, and project managers who actively create spaces for ECRs to engage and lead at different levels.

Multiple programs and initiatives exist on regional, national, sub-national, or institutional levels that support the career and skill development of ECRs. For example, the Marie Skłodowska-Curie Actions (MSCA) and the European Research Council (ERC) provide grants for ECRs to pursue innovative research and advance their academic careers. Additionally, MSCA’s Doctoral Networks programme (and the preceding Innovative Training Networks Programme) fosters collaboration by connecting ECRs with universities, research institutions, and the private and/or public sector. National initiatives, such as those by the Dutch Research Council (NWO), further support early career development through early career funding opportunities, such as the VENI grant for ECRs, and by organising VENI-awardee seminars and other networking opportunities enabling ECRs to learn from and connect with each other. Additional opportunities tailored for early-stage ECRs, particularly PhDs, are research schools or networks within and among universities and institutes, as well as summer schools (e.g., the Uppsala Summer School on Natural Hazards & Disaster Risk, co-organized by the Centre of Natural Hazards and Disaster Science). While much attention is given to individual career development in the European research landscape, guidance on how to empower ECRs within research projects remains limited.

Notwithstanding the aforementioned types of programmes, research projects rarely incorporate explicit strategies or guiding principles for empowering ECRs. Moreover, there is limited documentation on how such empowerment benefits both ECRs and the projects to which they contribute. This is a missed opportunity, given that many ECRs begin and advance their academic careers by working on such research projects, and these projects can offer invaluable opportunities and ideal conditions to develop key skills necessary for future roles.

**Figure 2.**
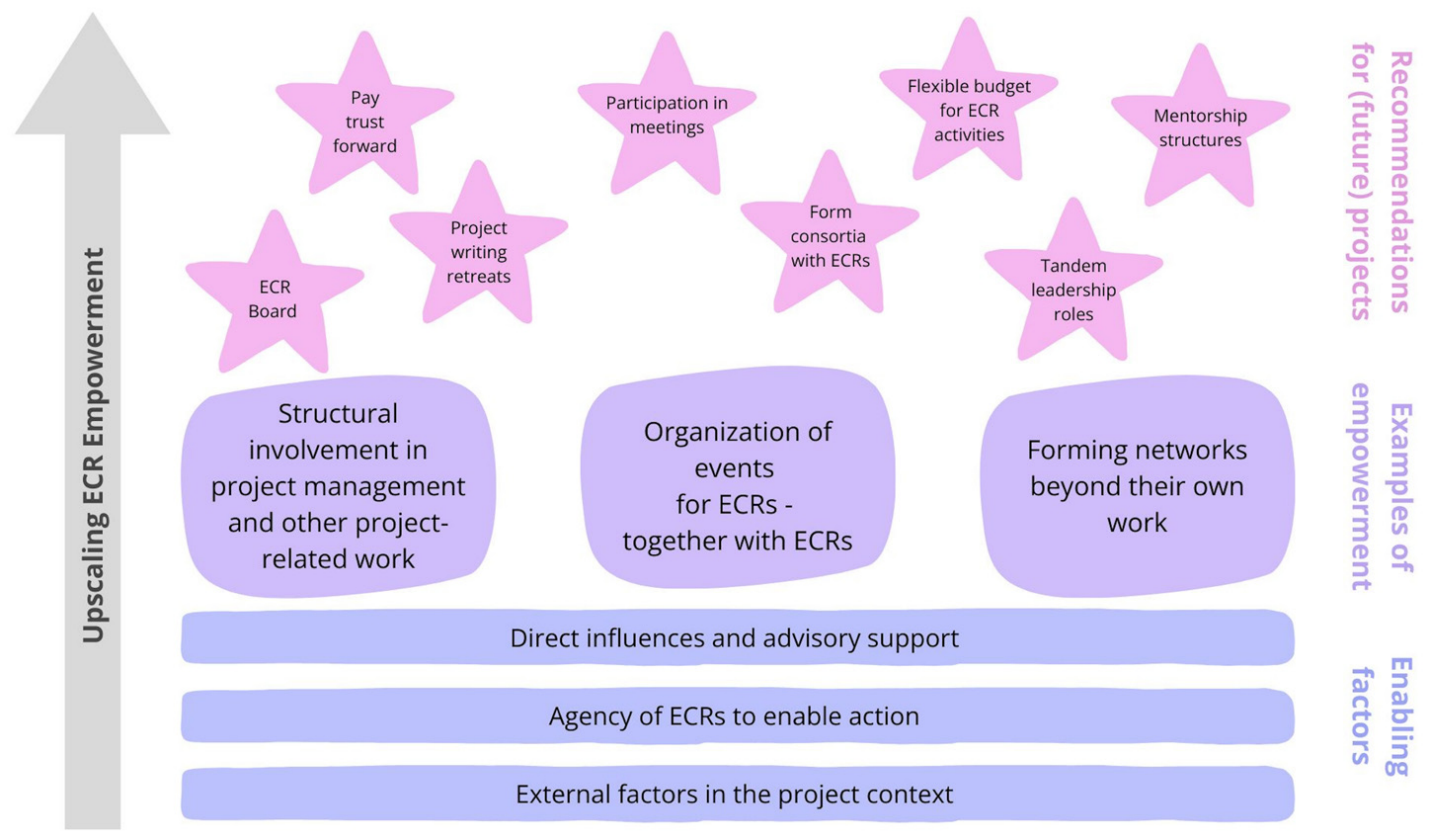
Graphical abstract. Pathways for embedding and upscaling ECR empowerment within research projects, illustrated and informed through experiences of the MYRIAD-EU project. Building upon broader enabling factors (blue bars), empowerment manifests in different ways (purple rectangles). Reflecting on these experiences and lessons learnt, recommendations are proposed to inform and inspire (future) projects (pink stars).

In this commentary, we provide examples of ECR empowerment in a European research project and reflect on key factors that enabled such empowerment ( Figure 2; blue bars). Additionally, we offer practical suggestions for how current and prospective projects can support ECR empowerment and engagement, from easy, low-hanging fruits to more ambitious initiatives ( Figure 2; purple rectangles and pink stars). We also share recommendations for funding agencies on how they can encourage and institutionalize such empowerment across research projects. We draw on perspectives and experiences of ECRs at different career stages as well as senior researchers. These insights were collected on multiple occasions and in various formats over the past four years, including informal conversations among ECRs and reflection sessions at various meetings (e.g., general assemblies) within the aforementioned project. We also shared and tested our ideas on key factors in a broader setting through a discussion session at the European Geosciences Union (EGU) in Vienna in April 2025.

## 2. Examples of promising empowerment from MYRIAD-EU and its benefits for the project and the ECRs

MYRIAD-EU ^1^ ( Ward *et al*., 2022) is a four-year, EU Horizon 2020-funded project with a consortium of 19 project partners from research and private sectors. Altogether, the project involved 133 participants, of which about 30% were considered ECRs at the beginning of the project (i.e., 30 early-stage ECRs and 11 late-stage ECRs). The project aimed to provide policymakers, decision-makers, and practitioners with practical tools to create forward-looking disaster (multi-)risk management strategies. Central to the project were pilot study teams that tested methods developed within the project through a collaborative co-design process with local stakeholders, addressing region- and sector-specific sustainability challenges.

According to feedback from the ECRs themselves, as well as from the project's External Advisory Board and Project Review Board, ECR engagement has been a key strength and outcome of the project. Based on repeated reflections and discussions among ECRs and with other project participants, three themes were identified as to how MYRIAD-EU accomplished the act of engaging and empowering ECRs. Firstly, the structural involvement of ECRs within project management and other deliverables. Secondly, allowing ECRs to design and organize events targeted at their career and personal development. Lastly, the emphasis and importance of forming networks for ECRs, outside of their direct work. Examples of ECR empowerment within these themes are discussed in the following sub-sections.

### 2.1. Theme 1: Structural involvement in project management and other project-related work

The primary means by which MYRIAD-EU structurally included ECRs in project management was by establishing the presence of an Early Career Researcher Board (ECRB) within the project proposal. The ECRB was integrated into the proposal, inspired by experiences from previous EU-funded projects, such as RECEIPT ^2^, where bottom-up ECR engagement organically led to the formation of similar bodies, yielding significant benefits for ECRs as well as the projects. The MYRIAD-EU ECRB was populated by self-volunteered ECRs from the consortium, with 4–5 ECRs of equal gender balance. A first ‘interim’ ECRB was installed at the start of the project for 6 months, to ensure that those who joined the project later had an opportunity to volunteer. Then, a second ECRB was installed for ~2 years, and a third ECRB for the final ~2 years. Each year, a new Chair of the ECRB, the so-called Early Career Representative, was elected by the members of the ECRB. Notably, the Early Career Representative was a full member of the project’s Management Team (MT). This rotation of ECRB members and the ECR Representative allowed for a large number of ECRs to become engaged in the management of the project.

Within the scope of MYRIAD-EU, the ECRB served as a primary contact point for all ECRs with the management team (MT). The position of the Early Career Representative allowed ECRs to actively participate in the processes of managing a research project that aimed to achieve societal impact, furthering their professional development. The ECRB also advocated the needs and concerns of all ECRs to the MT, for instance, resulting in active invitation across different career stages to join as co-conveners in the co-organized 3rd International Conference on Natural Hazards and Risks in a Changing World ( https://www.changingworldrisks2024.eu/). It also led to allocation of time at project meetings to reflect on the learnings on ECR empowerment and to share and receive feedback on their work, networking opportunities, and social activities. These sessions enabled ECRs to further develop their science communication skills and also kept the entire consortium informed about emerging research. Feedback from these sessions indicated that they were among the highlights of each General Assembly.

Another structural inclusion of ECRs was through the research activities and execution of the Work Packages (WPs), as well as the overall project management. While one senior researcher served as the principal investigator for the project, they worked in close collaboration with a late-stage ECR (e.g., post-doc) to address the financial, logistical, and reporting needs for the project. This set-up reduced the individual workload and offered both individuals new perspectives and opportunities to learn from each other. Furthermore, in the WPs, ECRs were primarily involved through contributions of developed methods, results, insights, and reflections to project deliverables. Importantly, later-stage ECRs were positioned as (co-)leads within their WPs, allowing them to deepen their experience with research management, reporting, meeting planning, and cross-partner collaboration. In one WP, the leadership was even offered to, and taken up by, an early-stage ECR at the end of their PhD. The WPs also benefited from advocacy for space for ECRs in regular work-package meetings. ECRs were encouraged to present and discuss their ongoing work, which allowed them to place their work within the broader research landscape and demonstrate the value of their contributions to the deliverables.

### 2.2. Theme 2: Organization of events for ECRs - together with ECRs

The MYRIAD-EU project also promised to organize multiple events. Amongst others, a scientific conference and a summer school. While the scientific conference was not purely an ECR-focused event, during the planning, the idea arose within the MYRIAD-EU MT to have an ECR sub-event as a component of the program. It was supported by the entire conference organising team to extend the conference by a set of ECR-specific events. As such, a team of early-stage ECRs from different projects formed to develop a program. The challenge of this ECR-day was that it had not been part of the original scoping and respective budget allocation, and thus faced financial constraints. However, the team of ECRs worked closely with the central organizing committee to find a solution. It was ultimately able to host an informal networking event attended by over 80 ECRs as well as a set of thematic excursions on the day after the conference, again offering an informal setting to connect. Opportunities like this provide an opportunity to deepen relationships with fellow researchers, get to know each other, and form personal connections that are beneficial when developing new research ideas or identifying suitable collaborators.

One of MYRIAD-EU’s commitments was to organize a summer school during the project, directly targeted towards ECRs. To accomplish this, MYRIAD-EU partnered with similar projects, including PARATUS ^3^, The HuT ^4^, and DIRECTED ^5^. Instead of a top-down approach, the MTs of the different projects entrusted ECRs to lead both the development and implementation process. After an open call to all ECRs from the four projects, a core team of five ECRs was assembled and directly supported by one senior researcher. A broader organizing team was established, including members of the MTs and additional ECRs, to ensure coordination and accountability. The ECR-led team reimagined the traditional summer school format. Rather than passive lectures, they designed an interactive and collaborative academy, where the agenda consisted of dynamic workshops, discussion forums, and problem-solving sessions ( https://drr-academy2024.cimne.com/). Additionally, participants actively contributed by leading their sessions and shaping the academy’s content.

An important component of the academy was a day-long workshop on proposal writing, which was included based on participant needs and curiosities, especially as early-stage ECRs are often underrepresented in proposal writing, despite the growing need to develop or get involved in winning proposals to secure (tenure) positions. Based on lessons and reflections shared in the workshop, participants were encouraged to develop their research proposals in response to a fictitious call. Some of these proposals even lead to the development of collaborative research projects between participants. The workshop provided a broad and open space for ECRs to approach more experienced researchers with their questions and gain experience with the dynamics and challenges of developing research proposals.

The approach to organizing an ECR-oriented event, which combines elements of both top-down and bottom-up approaches, benefited both the ECRs and the projects. The early-career organizers gained valuable experience in event management, collaboration, and decision-making. Meanwhile, participants who were empowered as session convenors strengthened their skills and expanded their networks to all those who attended. The projects, in turn, benefited from fresh, innovative formats and the increased capacity of engaged ECRs. The success of this event is evident in the way connections were formed, leading to new collaborative research initiatives and preparation for future editions of the academy.

### 2.3 Theme 3: Forming networks beyond their own work

Within MYRIAD-EU, ECRs actively engaged in various ways to form, extend, and engage with networks within the project and beyond. A significant amount of the networking was initiated during in-person project meetings, where ECRs had regular opportunities to engage with peers, senior researchers, and non-academic partners. Over time, these initial contacts evolved into both mutual support systems and mentoring, as well as extended professional networks. For example, ECRs began co-authoring manuscripts, co-convening conference sessions, organizing workshops, and initiating short-term research stays with partners they had met through the project. The trust and familiarity built through these early and repeated encounters also allowed them to benefit from the networks of the more established colleagues. For instance, more senior colleagues connected ECRs with valuable contacts from their networks (e.g., interviewees, stakeholders) that advanced and benefited specific research goals in addition to the development of the ECRs' professional networks.

Beyond the boundaries of project meetings, academic conferences played a key role in expanding networks. Informal networking events organized by ECRs during major scientific gatherings, such as the European Geosciences Union (EGU) General Assembly or the International Conference on Natural Hazards and Risks in a Changing World, provided familiar touchpoints with the networks and a space to bring in new contacts. These recurring informal “check-ins” helped maintain momentum and strengthened a sense of scientific community.

Additionally, ECRs from other research group structures, both inside and outside the project scope, had the opportunity to connect with fellow ECRs working on similar topics. For example, visiting researchers were invited to present during project meetings, join writing retreats, or contribute to project deliverables. These exchanges not only supported ECR skill-building and international exposure but also reinforced MYRIAD-EU’s visibility within the broader research landscape.

## 3. Key factors that enabled empowerment within MYRIAD-EU and beyond

When reflecting upon the three themes as outlined in the previous section in informal conversations, reflection sessions at the project meeting, or the short course at the European Geosciences Union General Assembly 2025, we identified a set of cross-cutting factors that are prerequisites or enabling factors for effective empowerment of ECRs ( Table 1): the direct influences and advisory support, the agency of the ECRs themselves, and a wide array of other external factors in the project set-up.

**Table 1.** Overview of key general factors for empowerment and encouragement, including selected sub-factors and the actors responsible for their implementation. PI: Principal Investigator.

General Factor	Sub-Factors	Actor(s)
**Direct influences and advisory support**	Good communication for the project deliverables	PI, ECR advisor
	Visibility of ECRs	PI, ECR advisor
	Support for skill development through shared learning and interdisciplinary exposure	PI, ECR advisor, non-ECR consortium members
	Early and public recognition of ECR (non-)scientific contributions	Project Management, ECR advisor
**Agency of ECRs to enable action**	Positively embracing professional development opportunities	ECR themselves
	Establishment of a peer-to-peer community	ECRs within the consortium
	Reflection on time-capacity and working capacity	ECRs themselves, ECR advisor
**Other factors in the project context**	Structure and distribution of ECRs across consortium partners	PI, Proposal writing team, Project Management
	Dynamics and interactions between ECRs and other project partners	Project Management, ECR advisor, Project Team
	Dedicated resources and flexible funding for ECR development	PI, Proposal writing team, Project Management

### 3.1 Direct influences and advisory support

One of the most critical enabling factors of strong empowerment and encouragement is the direct influence of advisory and supervisory support. Such support helped to foster trust and confidence among ECRs in their advisory relationships and their connection to the consortium, all of which are critical pillars of empowerment. It expressed itself in many ways, such as taking the time to share reflections on plans and experiences in similar situations, as well as offering suggestions and opportunities. Advisors can achieve this either by being proactively engaged or simply by being approachable.

Trust is built through engagement and shared responsibility, requiring both a commitment of time and the willingness of those established to pay the trust forward. Trust from advisors or supervisors means entrusting ECRs with control over the execution of project-related tasks or deliverables (e.g., through forming an ECRB, offering other opportunities, or sharing networks or knowledge). Trust is further built by active engagement with ECRs, repeatedly and continuously, especially at the beginning of the collaboration (e.g., through weekly meetings, (virtual) coffee chats). Additionally, transparent and honest communication is essential to building trust and confidence. Advisors and supervisors should provide clear communication of expectations, as well as constructive and truthful feedback. They should also take time to check-in, and exchange updates that are interesting to the ECR.

On the other hand, the ability to encourage, acknowledge, and guide is an essential attribute of a mentor to build confidence in ECRs. This requires initiative and a commitment to staying engaged from a distance, striking a careful balance between proactively offering advice, sharing personal experiences and observations, and remaining approachable so that ECRs feel comfortable seeking support when needed. In the MYRIAD-EU project, this was reflected in regular update meetings, complemented by the availability of senior colleagues for one-on-one conversations. Additionally, timely responses to requests for brief meetings or questions were perceived as valuable in fostering a strong sense of belonging and boosting the ECR's confidence. 

Being an effective advisor also means being open and inclusive. Acknowledging that ECRs are a diverse group of individuals involved in the research process can help tailor strategies to different experiences and empower individuals, for example, by acknowledging (non-)science activities that contribute to professional development. Finally, it is vital that project management, senior staff, and advisors are willing and motivated to attend ECR events, and vice versa. The participation of project partners in ECR-led and initiated events in MYRIAD-EU fostered mutual respect and the understanding that knowledge can be gained and shared at all career levels.

### 3.2 Agency of ECRs to enable action

Alongside a motivated and encouraging advisor, ECRs also need to possess an intrinsic motivation and agency, which the advisor can encourage and awaken. We want to underline that this is a highly individual trait, strongly influenced by the identity, socialization, experiences, and privileges of each ECR. Yet, based on the experiences in the MYRIAD-EU project, we believe that specific actions and intentions at varying degrees can already foster the empowerment of ECRs. One of these intentions is the willingness to step out of one's comfort zone, such as asking for attention and initiating conversations with seniors, standing by their beliefs or preferences regarding time allocation, and embracing (partial) failure as part of the learning process. Senior staff can teach these lessons through shared experiences of success, failure, and challenges.

ECRs should also have an idea of what they want to learn, contribute, and communicate this to their senior colleagues, advisors, and peers. In the context of MYRIAD-EU, support systems, such as informal get-togethers to share challenges, successes, and open questions, or bilateral conversations with other colleagues and friends, helped prioritize time allocation to different interests, develop ideas, and build confidence to stand by these ideas. They often served as the first level of receiving and responding to feedback and reflections from outside perspectives, preparing for more critical conversations with advisors or supervisors. Given that ECRs within MYRIAD-EU shared the same project goals, part of these support systems were embedded in the project context and thus offered additional contextualized peer-based support, such as opportunities for collaboration and co-promotion.

### 3.3 External factors in the project context

Additionally, external factors play an integral role through indirect influences or the setting and context of the research project. First of all, luck and chance are involved, as opportunities sometimes arise unexpectedly and spontaneously, and they are beyond one's control. It is the role of the advisor or more senior colleague to make ECRs aware of these opportunities and provide them equally/fairly. Along these lines, it also includes the compatibility of communication and working styles of ECRs, their advisors or supervisors, and other project partners.

Another essential factor is the availability of resources. Funding is crucial in determining participation, mobility, and available time. Additionally, having appropriate and flexible funding strategies and administrative support helps to ensure that essential resources are available and can be utilised to offer opportunities to ECRs as they progress through a project. At the same time, the funding influences the structure of the consortium, the people involved and the time they have distribution across the different required tasks, including mentoring of ECRs. In MYRIAD-EU, a pretty ambitious project with many promised deliverables and outcomes, the project relied on a substantive amount of PhDs, Post-Docs, and other ECRs spread across and affiliated with a few of the partners. It seemed that the structure of MYRIAD-EU offered the ‘right’ mix and allocation of different career stages across partners, which incentivised ECRs to establish a peer community for support, exchange, and collaboration. Again, this is strongly influenced by the personalities and interests of all involved partners. Still, we believe that structuring the project in a way that includes not too few but also not too many ECRs - also across the different ECR stages - contributed significantly to fostering an environment of lively collaboration, excitement, and engagement.

## 4. Looking ahead

Over the four years of the MYRIAD-EU project, we had ample opportunity to reflect on how ECR empowerment could be scaled up in future research collaborations. Here, we offer practical suggestions for how current and prospective projects can support ECR empowerment and engagement, from easy, low-hanging fruits to more ambitious initiatives. We also share recommendations for funding agencies on how they can encourage and institutionalize such empowerment across research projects.

### 4.1 Options to scale up empowerment in future projects

***Start small: give time and space (low-hanging fruit)***

Empowering ECRs doesn’t require a significant overhaul of project structures. Simple, intentional steps, such as giving ECRs active roles in WPs or representation in project management, can go a long way. Mechanisms like an **ECR board** or dedicated presentation slots at project meetings require little time or resources, but they signal that ECR voices matter. These structures create space for ECRs to actively reflect, speak up, and contribute to the strategic direction of a project. Importantly, they legitimize ECRs as researchers in their own right, ensuring their contributions are visible and valued.

Crucially, assigning ECRs a role in project management should come with a commitment to openness from senior colleagues. This openness allows ECRs to have a meaningful influence, even if they begin without formal leadership roles. The quality of their contributions will naturally vary depending on their level of engagement (same as any other work package lead or representative in the Management Team). Including such roles in project proposals requires the willingness to ‘pay forward’ due to an awareness and openness to challenge traditional project management.

***Mentorship and integration (medium effort)***

A slightly more involved but highly rewarding step is establishing consistent **mentorship structures**, particularly in the early stages of a project. As discussed in Section 3.1, supporting ECRs in understanding how their work fits into the broader research landscape can strengthen connections between tasks and lead to more integrated, impactful research.

An idea is the creation of **tandem leadership roles**: pairing a senior colleague with an ECR to co-lead work packages or project roles (e.g., ethics, data management). This approach requires extra coordination and time but provides additional resources and continuity, especially as team members move positions or take personal breaks. Less familiar with the ways of work package management, ECRs might offer fresh perspectives and creative ideas to organize and deliver. At the same time, ECRs gain hands-on experience in project leadership, an invaluable step toward becoming an independent researcher. Conversely, the ECR may come with particular expertise needed (e.g., in ethics), which means they can offer mentoring to someone else.

***Support community-building (low-hanging fruit to high effort)***

We noticed that efforts to encourage the formation of an ECR community within and beyond the project can further enhance ECR engagement and commitment to the project objectives. Projects should ensure that ECRs, along with senior colleagues, have the opportunity and budget to attend conferences and project meetings frequently. This is important for joint participation of all levels of experience at these events. This exposure enhances their understanding of the project’s broader context and enables them to bring new insights back to their work.

With greater efforts, projects can create additional dedicated spaces for ECR collaboration. One example is **across-project writing retreats** that enable ECRs from different project partners to gain a better understanding of what others are doing, develop ideas for collaboration, or enhance existing collaborations through in-person sprints. Another example could be the active encouragement of ECRs to participate in research visits at partner institutions or beyond. It offers another invaluable opportunity for collaboration and developing as an independent researcher.

Ultimately, most of these initiatives rely on having a dedicated budget for ECR-led or ECR-empowering activities. It all starts with forming project consortia, which have a sufficiently large number of ECRs in different career stages. Beyond that, budgets for activities might range from low-cost items, such as (local) conference participation, to medium-level efforts, like writing retreats, to higher-investment initiatives, such as research visits. The specific priorities of any given ECR community may vary by project and by the individual needs of those who make up the community. One approach to addressing this could be to allocate a specific budget more broadly for ECR activities, which could be further refined in the first year of a project in consultation with the ECRs.

### 4.2 Ideas on how funding bodies could incentivize empowerment within projects

Ultimately, funders shape the incentives and requirements that drive project behavior. If structural ECR empowerment is to become a norm rather than an exception in research projects, funding bodies must take an active role.

Some funding instruments, such as the EU’s doctoral networks, explicitly incentivise the empowerment of ECRs, while others do this less explicitly. These latter funding opportunity types could encourage ECR empowerment by explicitly incorporating it in proposal templates and evaluation criteria. Of course, to make this actionable, project funding mechanisms should allow for the proposed empowerment activities. If ECR empowerment is included in evaluation criteria, with adequate funding, an impactful step for funders would be to include ECR empowerment in annual reporting templates. Even a few open-ended prompts, such as *“How has the project supported ECR involvement in management?”* or *“What strategies were used to support ECRs in becoming independent and critical researchers?”*, could be low-effort additions. Being asked to report on such questions will help project managers consider these aspects repeatedly. More structured reporting (e.g., project-wide surveys) could help surface both barriers and success stories, potentially benefiting other (underrepresented) groups as well.

Creating or promoting targeted, centralized platforms for ECR networks across funded projects could also have lasting benefits. While we attempted within MYRIAD-EU to set up a research-focused, specific active ECR network, we faced structural constraints and limited engagement. At the same time, many platforms by funding bodies such as the European Commission already exist, but ECRs or project managers might not be aware of the most relevant ones. Funding bodies could offer guidance, experience, and support to project managers and ECRs, helping ECRs share experiences, exchange research content, and access guidance and resources. This ultimately amplifies the collective impact of publicly funded science.

## Disclaimer

The views expressed in this article are those of the author(s). Publication in Open Research Europe does not imply endorsement of the European Commission.

## Ethics and consent

Ethical approval and consent were not required.

## Data Availability

No data are associated with this article.
