# Supplementary material for: Empowering ECRs to make research projects flourish: lessons from a European research project
Source: Open Res Eur. 2026 Aug 3;5:312. Originally published 2025 Oct 15. [Version 3] doi: 10.12688/openreseurope.21517.3 (PMC13191317; doi:10.12688/openreseurope.21517.3)
Supplement: Peer review history

# Reviewer report: Reviewer response for version 3

**DOI:** 10.21956/openreseurope.26751.r78492

**Published:** 2026-08-12

**Authors:** Marleen Wierenga

**Affiliations:** 1 Norwegian University of Science and Technology, Trondheim, Norway

Thank you for the revised version of the article. I have indeed been able to assess it and I believe the revision is of an acceptable scientific standard and would like to approve it.

Is the case presented with sufficient detail to be useful for teaching or other practitioners?

No

Is the work clearly and accurately presented and does it cite the current literature?

Yes

If applicable, is the statistical analysis and its interpretation appropriate?

Not applicable

Are all the source data underlying the results available to ensure full reproducibility?

No

Are the conclusions drawn adequately supported by the results?

Partly

Is the background of the case’s history and progression described in sufficient detail?

Yes

Reviewer Expertise:

NA

I confirm that I have read this submission and believe that I have an appropriate level of expertise to confirm that it is of an acceptable scientific standard.

# Reviewer report: Reviewer response for version 2

**DOI:** 10.21956/openreseurope.25625.r73720

**Published:** 2026-07-15

**Authors:** Marleen Wierenga

**Affiliations:** 1 Norwegian University of Science and Technology, Trondheim, Norway

Thank you for the opportunity to read your article. In my reading, this case study focused on ways to empower early-career researchers. I support the effort and agree that ECRs can carry responsibilities in large research projects. I have two comments that relate to each other which I would like to elaborate on below.

 Yet, as your definition also states, PhD students (which the actors in the case study primarily are) are individuals in a stage of becoming and they are becoming researchers. This links to my main concern on whether engagement in project management, organizing events and other project-related tasks leads to unintended consequences, such as feeling overwhelmed, burnout or delay in finishing PhD studies. As a PhD without project-related tasks can be overwhelming, having additional responsibilities can make it even more overwhelming.

 Additionally, PhD-supervisor relationships can be asymmetrical as the supervisor has more power and resources. When discussing the empowerment of PhD students, one should be aware of the risk for exploitation. For example, in Theme 1 where PhD students are involved in project management, I am wondering if these are substance-related or administrative tasks. The first develop substance-expertise and project management skills while the latter could be seen as tasks someone higher in the hierarchy outsources to a PhD student, who does not learn much from those tasks, but it is nevertheless time away from one’s research.

 In Theme 2, I am wondering what the compensation for the PhD students organizing events is, considering that event organizing is not a core part of “becoming an independent researcher”.

 Taking these two comments together, it might be useful to include a reflection on boundary conditions under which the practices you suggest can be successful. One boundary condition seems to be supportive supervisor and/or advisor, but others could be related to institutional conditions (e.g. overtime compensation, PhD extension policies, etc.).

 A final minor comment is that Figure 1 and 2 seemed to have changed places: what you refer to as Figure 1 is actually Figure 2. I would double-check that you have the correct figures with the correct labels.

  

- Are all the source data underlying the results available to ensure full reproducibility?

 No, as the manuscript is about self-reflection on the role different actors played during the implementation of the project, this does not seem to be a study aimed for reproducibility making data availability less relevant.

- Are the conclusions drawn adequately supported by the results?

Partly:  I have some remarks that are addressed in the report above.

- Is the case presented with sufficient detail to be useful for teaching or other practitioners?

No: The target audience for the manuscript seems to be project managers, and not teachers or practitioners.

Is the case presented with sufficient detail to be useful for teaching or other practitioners?

No

Is the work clearly and accurately presented and does it cite the current literature?

Yes

If applicable, is the statistical analysis and its interpretation appropriate?

Not applicable

Are all the source data underlying the results available to ensure full reproducibility?

No

Are the conclusions drawn adequately supported by the results?

Partly

Is the background of the case’s history and progression described in sufficient detail?

Yes

Reviewer Expertise:

NA

I confirm that I have read this submission and believe that I have an appropriate level of expertise to confirm that it is of an acceptable scientific standard, however I have significant reservations, as outlined above.

# Response

**Published:** 2026-07-22

**Authors:** Kelley De Polt

**Reviewer Comment 4.1:** Thank you for the opportunity to read your article. In my reading, this case study focused on ways to empower early-career researchers. I support the effort and agree that ECRs can carry responsibilities in large research projects. I have two comments that relate to each other which I would like to elaborate on below.  

**Author Response 4.1:** We thank the reviewer for their time and thoughts invested in this manuscript. We are glad the reviewer finds this article relevant and overarchingly in alignment with their own experience and expertise. We respond to the comments below and also added a tracked-changes version of the manuscript.  

**Reviewer Comment 4.2:  **Yet, as your definition also states, PhD students (which the actors in the case study primarily are) are individuals in a stage of becoming and they are becoming researchers. This links to my main concern on whether engagement in project management, organizing events and other project-related tasks leads to unintended consequences, such as feeling overwhelmed, burnout or delay in finishing PhD studies. As a PhD without project-related tasks can be overwhelming, having additional responsibilities can make it even more overwhelming.  

**Author Response 4.2: **We agree with the reviewer about the challenges of expectations towards PhDs. We made two clarifications in the text, one to make explicit what we understand under becoming an independent researcher, namely building competencies beyond ‘doing research’ including ‘working with others’ and ‘managing research’ as presented by the European Commission. The second modification clarifies the challenging and pressuring expectations:

- Introduction (2nd paragraph): “The early career stage is a critical period of transition from a dependent to an independent researcher, during which ECRs develop and expand their skill set ( Laudel & Gläser, 2008; Nicholas et al., 2019). According to the European Commission, the skills go beyond “doing research” and include other competencies such as “managing research”, or “working with others” (European Union, 2025). ECRs can be defined in different ways, but in this perspective, we use the European Geosciences Union definition, which defines ECRs as students, PhD candidates”
- Section 4.3 (2nd paragraph): “Therefore, it is crucial to have appropriate and flexible funding strategies in place, accompanied by appropriate levels of administrative support. For example, similar to some institutional arrangements which extend contracts for substantial teaching arrangements, project budgets or institutional arrangements could be set up to allow for extensions of PhD contracts for substantial work that is currently not counted towards their evaluation as a PhD candidate. This ensures that essential resources are accessible and can be utilised to provide opportunities for ECRs as they progress through a project. The more fundamental solution would be to align expectations regarding the outputs of PhD’s with the recommended competencies of researchers (European Union, 2025): if it is desired that researchers have skills to mobilize resources, manage projects, promote inclusion and diversity themselves, it might be worth being reflected in the evaluation criteria for completing a PhD. Instead of adding additional tasks on top, it would allow to re-allocate existing resources accordingly.”

 
**Reviewer Comment 4.3: ** Additionally, PhD-supervisor relationships can be asymmetrical as the supervisor has more power and resources. When discussing the empowerment of PhD students, one should be aware of the risk for exploitation. For example, in Theme 1 where PhD students are involved in project management, I am wondering if these are substance-related or administrative tasks. The first develop substance-expertise and project management skills while the latter could be seen as tasks someone higher in the hierarchy outsources to a PhD student, who does not learn much from those tasks, but it is nevertheless time away from one’s research.  

**Author Response 4.3:** We thank the reviewer for this comment. In line with our earlier modification defining what we understand under becoming an independent researcher, we believe that both research-related as well as administrative tasks can be useful ways to build competencies. We agree that exploitation in asymmetric relations is a risk always, which we point out explicitly in section 4.3, and also implicitly discuss in the context of the agency of ECRs.  

**Reviewer Comment 4.4: **In Theme 2, I am wondering what the compensation for the PhD students organizing events is, considering that event organizing is not a core part of “becoming an independent researcher”.  

**Author Rresponse 4.4:** We thank the reviewer for this comment. In line with our earlier modification defining what we understand under becoming an independent researcher, we believe that both research-related as well as administrative tasks can be useful ways to build competencies. We added an example of a possible flexible financing mechanism that could compensate for substantial efforts that are not considered core to the PhD, e.g. similar to teaching arrangements (see RC4.2).  

**Reviewer Comment 4.5:** Taking these two comments together, it might be useful to include a reflection on boundary conditions under which the practices you suggest can be successful. One boundary condition seems to be supportive supervisor and/or advisor, but others could be related to institutional conditions (e.g. overtime compensation, PhD extension policies, etc.).  

**Author Response 4.5: **We thank the reviewer for this suggestion. In addition to the earlier introduced change regarding the institutional, financial boundary conditions (see RC4.2), we also made the following modification:

- Section 4.3 (1st paragraph): “Along these lines, this external factor also encompasses the compatibility of communication and working styles between ECRs, their advisors or supervisors, and other project partners, as discussed above in section 4.1 as well as the underlying motivation and capacities of supervisors and advisors to fulfill and tailor their supportive role to each ECR.”

 
**Reviewer Comment 4.6:** A final minor comment is that Figure 1 and 2 seemed to have changed places: what you refer to as Figure 1 is actually Figure 2. I would double-check that you have the correct figures with the correct labels.  

**Author Response 4.6:** Thank you for noting this. This misalignment in figure references and positions occurred during production processing of the prior submission at the journal. We have corrected the figure labels and their positions in our resubmission and will remain attentive to this in case the issue occurs again.

# Reviewer report: Reviewer response for version 2

**DOI:** 10.21956/openreseurope.25625.r73519

**Published:** 2026-05-18

**Authors:** Leanne Gibbs

**Affiliations:** 1 School of Education, Charles Sturt University, Bathurst, New South Wales, Australia

Thank you to the authors for thoroughly and thoughtfully addressing all reviewer feedback, including my own and that of the other reviewers. Your revisions have significantly strengthened the manuscript, and it is now a clear, well-argued, and engaging Open Letter.

 I greatly appreciate the care taken in responding to the feedback and the comprehensive way in which the suggestions have been incorporated.

 I am very pleased to say that I have no further comments and recommend the Open Letter for indexing.

Reviewer Expertise:

Leadership in ECEC, ECRs, Research groups

I confirm that I have read this submission and believe that I have an appropriate level of expertise to confirm that it is of an acceptable scientific standard.

# Reviewer report: Reviewer response for version 1

**DOI:** 10.21956/openreseurope.23275.r62367

**Published:** 2026-01-10

**Authors:** Liudvika Leišytė

**Affiliations:** 1 TU Dortmund University, Dortmund, North Rhine-Westphalia, Germany

The article provides an important contribution in helping understand the way a collaborative international research project is organized and how it can support the empowerment of early career researchers. The initiatives undertaken in the project implementation to foster the voice of early career researchers seem to have been influential and the recommendations derived from the study are highly relevant.

 At the same time, the question is - what is the research problem that the authors aim to address? It needs to be better spelled out.

 The empirical base of the paper includes perspectives and experiences of ECRs and senior researchers involved in the project implementation. It would be important to indicate to the readers, which disciplines were researched in the study? At the moment the findings seem to be generalised, but we know that there are different research cultures, which imply different epistemologies and ways of working (e.g. Becher (1989) on Tribes and Territories of different disciplines).

 This means, that the findings may be applicable only to specific disciplines that were studied and particular type of collaborative project settings. This needs to be addressed in the methodology as well as discussion sections.

 When discussing the success of the initiatives undertaken for empowerment of ECRS more empirical evidence needs to be provided, for instance, a few examples of new collaborative research initiatives would be helpful.

 The limitations of the study need to be elaborated. What about the possible biases of the authors researching project implementation?

 Please clarify what is meant by „it“ in the sentence „Along these lines, it also includes the compatibility of communication and working styles of ECRs, their advisors or supervisors, and other project partners.“

Where applicable, are recommendations and next steps explained clearly for others to follow? (Please consider whether others in the research community would be able to implement guidelines or recommendations and/or constructively engage in the debate)

Partly

Does the article adequately reference differing views and opinions?

Yes

Are all factual statements correct, and are statements and arguments made adequately supported by citations?

Partly

Is the rationale for the Open Letter provided in sufficient detail? (Please consider whether existing challenges in the field are outlined clearly and whether the purpose of the letter is explained)

Yes

Is the Open Letter written in accessible language? (Please consider whether all subject-specific terms, concepts and abbreviations are explained)

Yes

Reviewer Expertise:

NA

I confirm that I have read this submission and believe that I have an appropriate level of expertise to confirm that it is of an acceptable scientific standard, however I have significant reservations, as outlined above.

# Response

**Published:** 2026-04-13

**Authors:** Kelley De Polt

RC - Reviewer Comment        A - Author Response

 TR - Text Revision

 Report 3: 

 (Liudvika Leišytė, TU Dortmund University, Dortmund, North Rhine-Westphalia, Germany; link)

 —   RC3.1:

 The article provides an important contribution in helping understand the way a collaborative international research project is organized and how it can support the empowerment of early career researchers. The initiatives undertaken in the project implementation to foster the voice of early career researchers seem to have been influential and the recommendations derived from the study are highly relevant.  

**AR3.1**: We thank this reviewer for taking the time to read and provide feedback on our manuscript. We are grateful for the recognition of the importance of the paper and have addressed the comments of all reviewers as outlined in our responses to further improve the manuscript. Additionally, we improved the readability and clarity of the text by some minor textual adjustments throughout the paper. The main modifications are as follows:

 We changed the format of the study to a case study framing to better align with the journal guidelines and better capture the scope and narrative of the manuscript.

 We added many additional references throughout the manuscript to better place our findings in the broader context of the scholarship. 

 We added critical reflections on the insights produced, notably by adding a positionality statement.

 In the following, we offer point-by-point responses. Responses to the review are written in a red font and numbered as AR (author response). Modifications in the text (TR, textual response) are marked in a blue font. In cases where smaller modifications within an existing paragraph are made, the changes are underlined; in cases where a new paragraph or section is added, we refrained from underlining for the sake of readability.

 —   RC3.2:

 At the same time, the question is - what is the research problem that the authors aim to address? It needs to be better spelled out.  

**AR3.2: ** In this manuscript we want to explicitly reflect and share experiences on the topic of ECR empowerment in research projects. As such, we approach it from a very practical and lived-experience angle to share an account from one project that explicitly had the topic of ECR empowerment on its agenda. We agree that in the earlier version of the manuscript, this was not fully clear, which is why we adjusted the article format to a case study paper. As part of that change, we adjusted Section 1. Introduction (TR3.2) to better introduce the research problem.   

 TR3.2:

 1. Introduction

 [...] In this study, we reflect how a research project, that explicitly accounts for the interests and needs of ECRs, effectively contributes to ECR empowerment and how it benefits from these efforts. We provide examples of ECR empowerment in a European research project and reflect on key factors that enabled such empowerment. Finally, we offer practical suggestions for how current and prospective projects can support ECR empowerment and engagement, from easy, low-hanging fruits to more ambitious initiatives. We additionally share recommendations for funding agencies on how they can encourage and institutionalize such empowerment across research projects.

 —   RC3.3:

 The empirical base of the paper includes perspectives and experiences of ECRs and senior researchers involved in the project implementation. It would be important to indicate to the readers, which disciplines were researched in the study?   

**AR3.3: **We thank the reviewer for this critical suggestion. As part of a new section (TR3.3) describing the case study and data collection, we also add a statement on the positionality, which includes reference to the represented disciplines, team dynamics and (ongoing) dependencies to give the reader a better view of the author team and potential biases.    TR3.3: 2. The case of MYRIAD-EU

 MYRIAD-EU (Ward et al., 2022) was a four-year, EU Horizon 2020-funded project with a consortium composed of 19 project partners from universities, research institutions, NGO, and the private sector. The project aimed to provide policymakers, decision-makers, and practitioners with practical tools to create forward-looking disaster (multi-)risk management strategies. Central to the project were pilot study teams that tested methods developed within the project through a collaborative, co-design process with local stakeholders, addressing region- and sector-specific sustainability challenges.

 In order to maximize the impact of the project, one of the measures implemented was to ensure a “prominent role for Early Career Researchers within the management structure” as promised in the project proposal. Consequently, the representation of ECRs and the explicit consideration of their interests, ideas and needs were viewed as a critical component of the project’s success. As a result, the project offers a promising step towards effective ECR empowerment that benefits both the ECRs and the project, making it a valuable and relevant case to study and reflect upon. Altogether, the project involved 133 participants, of which about 30% were considered ECRs at the beginning of the project (i.e., 30 early-stage and 11 late-stage). Most ECRs were employed as researchers from research institutes (n=17), PhDs (n=12), or Post-Docs (n=6) or tenure track positions at Universities (n=6) who had a primary role to contribute in the scientific work packages of MYRIAD-EU to develop and test new methods and tools or other deliverables and milestones. Some of the late-stage ECRs have been involved in the proposal writing for MYRIAD-EU and took on management responsibilities for specific work packages or the project as a whole.    2.1. The process of collecting learnings and reflections

 In the case study we draw on perspectives and experiences of ECRs at various career stages, as well as those of senior researchers. These insights were collected on multiple occasions and in various formats over the past four years, including informal conversations among ECRs and reflection sessions at various meetings (e.g., general assemblies) within the aforementioned project context. Additionally, we shared and validated our ideas on examples and key factors in a broader context through a short course at the European Geosciences Union (EGU) General Assembly (EGU25; Vienna, Austria) in April 2025.   Since the beginning of MYRIAD-EU, an active network has been established among the ECRs. Prior to each project general assembly, they organized a dinner together to connect, share experiences, and discuss project progress, their work, amongst other things. Additionally, they held occasional online check-in talks, roughly once or twice a year, with the explicit purpose of sharing recent successes or challenges, providing support, and brainstorming ideas for upcoming project activities. These meetups established trust among ECRs and provided the ability to share experiences as an ECR navigating their career path. Although no formal notes had been taken during these dinners or informal meetings, they collectively shaped a picture of the state of ECRs in the project.    Additionally, the ECRs organized a 2-hour workshop in the third year of the project. This workshop provided an opportunity to collect reflections on past experiences as ECRs in the project. They noted aspects they appreciated most, reflections they had regarding their role in the project, and aspects they thought would like to see changed, whether by themselves or by others in the project. The workshop also included a reflection on the remaining time in the project, where ECRs could share their plans, hopes, and wishes regarding their roles in the project, things to learn or to be involved in.    To validate key examples identified by the ECRs themselves in their own workshop, a session at the final project general assembly was organized which engaged with the entire project consortium to reflect on the involvement of ECRs in the project. The session aimed to collect instances where ECR involvement was noticed and which positive or unexpected experiences were made. Project partners were also encouraged to suggest ideas for future empowerment. During this session, project partners had the opportunity to comment on and reflect upon the notes contributed by others.  Additionally, they were tasked with ranking the collected aspects in terms of their perceived importance, ensuring these insights aligned with the broader consortium perspectives.   2.2. Positionality

 Our background and experiences shape the knowledge we produce as researchers (see e.g., Hausermann & Adomako, 2022). We are a team of co-authors working in disaster risk research, with disciplinary foundations in engineering or the natural sciences and varying experience in qualitative and quantitative methodologies. All authors were directly involved in the implementation of the MYRIAD-EU project and associated activities. We therefore write from embedded positions: as ECRs ourselves, as project managers or advisors responsible for meeting the project’s ambitions, and as members of an ECR board tasked with representing ECR interests in the management processes. Many of us are or were affiliated with the coordinating institution, which may shape how we interpret and present the project’s outcomes.    The study did not follow a predefined qualitative research design, nor was it conducted by an external team independent of the project’s internal hierarchies and dependencies. As such, academic power dynamics, institutional affiliations, professional relationships and the disciplinary context may have influenced both how experiences were expressed and how they are interpreted in this study. During the writing process, individual accounts were compared and discussed among co-authors in an effort to surface differing perspectives and critically question our interpretations. Nevertheless, the conclusions presented remain shaped by our shared disciplinary background, professional interdependencies, and collective investment in the project’s success. As a result, we focus on promising empowerment examples rather than questioning what could have gone better. This might already be a very important learning and insight for new projects to take into account.   

 —   RC3.4: 

 At the moment the findings seem to be generalised, but we know that there are different research cultures, which imply different epistemologies and ways of working (e.g. Becher (1989) on Tribes and Territories of different disciplines).   This means, that the findings may be applicable only to specific disciplines that were studied and particular type of collaborative project settings. This needs to be addressed in the methodology as well as discussion sections.  

**AR3.4:** As outlined in AR3.3, we added a positionality statement as part of the methods (see TR3.3) and have also refined the introduction of section 5. Looking ahead to explicitly mention this limitation.   

 TR3.4: 5. Looking ahead

 Over the four years of the MYRIAD-EU project, we had ample opportunity to reflect on how ECR empowerment could be scaled up in future research collaborations. Our reflections, shaped by our shared disciplinary backgrounds, professional interdependencies, and collective investment in the project’s success, are inherently biased. Furthermore, as the project had the broad intention to strengthen ECR empowerment, it did not include specific targets or objectives that could be used to compare the effectiveness or success of specific discussed measures against. Yet we believe they offer valuable and transferable insights that could be taken on-board in or compared with findings from future projects. [...]  

 —   RC3.5: 

 When discussing the success of the initiatives undertaken for empowerment of ECRS more empirical evidence needs to be provided, for instance, a few examples of new collaborative research initiatives would be helpful.  

**AR3.5: **We have enhanced our discussion by incorporating specific examples to illustrate the outcomes and follow-up activities of these initiatives. In the Theme 2 section (TR3.5), we have added examples of collaborative projects that have emerged from activities such as the ECR academy. For instance, papers have been published highlighting the fruitful collaborations between ECRs and senior researchers. Additionally, conference sessions organized by ECRs have attracted significant interest and led to further opportunities, particularly networking. Additionally, notably, the ECR academy was successfully hosted again in 2026. These outcomes underscore the sustained effort and continuity of our initiatives, which are essential for fostering a supportive empowerment of ECRs.   TR3.5: 3.2. Theme 2: Organization of events for ECRs - together with ECRs

 Over time, these initial contacts evolved into both mutual support systems and mentoring, as well as extended professional networks. For example, ECRs began co-authoring manuscripts (e.g., Article in Iscience; Tiggeloven and Pfeiffer 2025), co-convening conference sessions (e.g., EGU 2026 Session: Early Warning Systems (EWS): From Science to Action for Effective Disaster Risk Reduction), organizing workshops (e.g. short course on ECR empowerment at EGU 2024 and 2025), and initiating short-term research stays with partners they had met through the project. Another example is the concept of the DRR Academy which was carried forward and refined in a 2nd edition in 2025 by a newly composed group of early-career organisers.  

 —   RC3.6: 

 The limitations of the study need to be elaborated. What about the possible biases of the authors researching project implementation?  

**AR3.6**: As discussed in AR3.3, we have added a positionality statement to explicitly address the potential biases of the authors involved in the research and analysis. Additionally, the text discussing the forward-looking aspects has been adapted to incorporate this perspective, thereby reinstating our positioning within the broader context. Furthermore, we have highlighted how our findings have been validated and aligned with those of the broader geoscience domain.  

 —   RC3.7: 

 Please clarify what is meant by „it“ in the sentence „Along these lines, it also includes the compatibility of communication and working styles of ECRs, their advisors or supervisors, and other project partners.“  

**AR3.7**: We adjusted the section discussing external factors to enhance clarity. Thank you for pointing out the grammatical inconsistencies. We intended "it" to refer to the compatibility of communication and working styles mentioned earlier. The section has been revised accordingly, as outlined in TR3.7 below.  

 TR3.7:     4.3 External factors in the project context

 Along these lines, this external factor also encompasses the compatibility of communication and working styles between ECRs, their advisors or supervisors, and other project partners, as discussed above in section 4.1. —

# Reviewer report: Reviewer response for version 1

**DOI:** 10.21956/openreseurope.23275.r66630

**Published:** 2026-01-07

**Authors:** Kofi Agyekum

**Affiliations:** 1 Kwame Nkrumah University of Science and Technology, Kumasi, Ghana

The paper is an Open Letter reflecting on how Early Career Researchers (ECRs) can be meaningfully empowered within large, collaborative European research projects. It draws basically on experiences from the MYRIAD-EU Horizon 2020 project. The paper argues that although ECRs constitute a substantial proportion of the research workforce, explicit strategies for their empowerment within projects is still underdeveloped.

 Critically examining this manuscript, certain key strengths are evident. Its focus on ECR empowerment is highly relevant given current concerns about academic precarity, research culture, and workforce sustainability. Also, it offers strong, practice-based examples rather than abstract recommendations. Another key strength observed is that it is well-organized around themes, enabling factors, and offers forward-looking recommendations. The paper offers a balanced recognition of various multiple actors. It acknowledges the fact that empowerment is not only the responsibility of ECRs, but also of principal investigators, supervisors, project managers, and funders.

 Notwithstanding these key strengths, various flaws exist that could be addressed to further improve it. The paper overly relies on one single case, i.e., the MYRAID-EU. This weakens the generalizability of the findings and makes it read more like a report from this project rather than a policy-oriented open letter. Another flaw is the fact that the core themes in the topic are not strongly underpinned by empirical or theoretical work. The nature of evidence presented is quite ambiguous. For instance, although the paper states that insights were gleaned from reflections, etc., it does not clarify how these reflections were gathered and analysed. Finally, this does not read more like an open letter as indicated earlier. It is more of a reflective case study.

 Certain key areas could be improved to better position this manuscript to contribute to the body of knowledge. Please consider strengthening its alignment with the format for an open letter. The core themes (e.g., ECR Boards, Agency, Mentorship, Networking, etc.) could be well-situated within the current state-of-the-art. The nature and scope of the reflections could be further clarified.

Where applicable, are recommendations and next steps explained clearly for others to follow? (Please consider whether others in the research community would be able to implement guidelines or recommendations and/or constructively engage in the debate)

Yes

Does the article adequately reference differing views and opinions?

No

Are all factual statements correct, and are statements and arguments made adequately supported by citations?

Partly

Is the rationale for the Open Letter provided in sufficient detail? (Please consider whether existing challenges in the field are outlined clearly and whether the purpose of the letter is explained)

Yes

Is the Open Letter written in accessible language? (Please consider whether all subject-specific terms, concepts and abbreviations are explained)

Yes

Reviewer Expertise:

Sustainable construction, net-zero construction, green buildings

I confirm that I have read this submission and believe that I have an appropriate level of expertise to confirm that it is of an acceptable scientific standard, however I have significant reservations, as outlined above.

## References

[1] : Empowering ECRs to make research projects flourish: lessons from a European research project. *Open Research Europe* .2025;5: 10.12688/openreseurope.21517.1 10.12688/openreseurope.21517.1 42182981 PMC13191317

# Response

**Published:** 2026-04-13

**Authors:** Kelley De Polt

Legend:

 RC - Reviewer Comment        A - Author Response

 TR - Text Revision

 Report 2: 

 (Kofi Agyekum, Kwame Nkrumah University of Science and Technology, Kumasi, Ghana; link) —

 RC2.1:

 The paper is an Open Letter reflecting on how Early Career Researchers (ECRs) can be meaningfully empowered within large, collaborative European research projects. It draws basically on experiences from the MYRIAD-EU Horizon 2020 project. The paper argues that although ECRs constitute a substantial proportion of the research workforce, explicit strategies for their empowerment within projects is still underdeveloped. Critically examining this manuscript, certain key strengths are evident. Its focus on ECR empowerment is highly relevant given current concerns about academic precarity, research culture, and workforce sustainability. Also, it offers strong, practice-based examples rather than abstract recommendations. Another key strength observed is that it is well-organized around themes, enabling factors, and offers forward-looking recommendations. The paper offers a balanced recognition of various multiple actors. It acknowledges the fact that empowerment is not only the responsibility of ECRs, but also of principal investigators, supervisors, project managers, and funders.

**AR2.1:** We thank the reviewer for their thorough summary and positive words about our manuscript. We have addressed the comments of all reviewers as outlined in our responses. Additionally, we improved the readability and clarity of the text by some minor textual adjustments throughout the manuscript. The main modifications are as follows:

 We changed the format of the study to a case study framing to better align with the journal guidelines and better capture the scope and narrative of the manuscript.

 We added many additional references throughout the manuscript to better place our findings in the broader context of the scholarship. 

 We added critical reflections on the insights produced, notably by adding a positionality statement.

 In the following, we offer point-by-point responses. Responses to the review are written in a red font and numbered as AR (author response). Modifications in the text (TR, textual response) are marked in a blue font. In cases where smaller modifications within an existing paragraph are made, the changes are underlined; in cases where a new paragraph or section is added, we refrained from underlining for the sake of readability. —

 RC2.2: 

 Notwithstanding these key strengths, various flaws exist that could be addressed to further improve it. The paper overly relies on one single case, i.e., the MYRAID-EU. This weakens the generalizability of the findings and makes it read more like a report from this project rather than a policy-oriented open letter. 

**AR2.2**: We thank the reviewer for this comment. Indeed, as this and other reviewers have noted, the paper refers and reflects mostly to one project which seems unsuited for an Open Letter format. As a result, we decided to adjust the format to one of a case study paper. Open Research Europe describes case study publications as those which “[describe] actual interventions or experiences”. And they “accept case studies examining a person, place, event, phenomenon, or other type of subject of analysis in order to highlight key themes”. In our paper, we primarily present the case of MYRIAD-EU sharing reflections and lessons from the different ways ECR empowerment took place and how it benefitted both the ECRs and the project. As such a case study format seems to match the requirements by Open Research Europe. At the same time, our study is not a typical case study experiment which can easily be reproduced. It is rather a unique account of a specific, complex process and its outcomes which is why we originally opted for an Open research letter.  In order to adjust the format, we add a new section 2 (see TR2.2) outlining the case study, the method for data collection and the data collection events themselves along with a statement on the positionality of our findings. TR2.2:

 2. The case of MYRIAD-EU

 MYRIAD-EU (Ward et al., 2022) was a four-year, EU Horizon 2020-funded project with a consortium composed of 19 project partners from universities, research institutions, NGO, and the private sector. The project aimed to provide policymakers, decision-makers, and practitioners with practical tools to create forward-looking disaster (multi-)risk management strategies. Central to the project were pilot study teams that tested methods developed within the project through a collaborative, co-design process with local stakeholders, addressing region- and sector-specific sustainability challenges.

 In order to maximize the impact of the project, one of the measures implemented was to ensure a “prominent role for Early Career Researchers within the management structure” as promised in the project proposal. Consequently, the representation of ECRs and the explicit consideration of their interests, ideas and needs were viewed as a critical component of the project’s success. As a result, the project offers a promising step towards effective ECR empowerment that benefits both the ECRs and the project, making it a valuable and relevant case to study and reflect upon. Altogether, the project involved 133 participants, of which about 30% were considered ECRs at the beginning of the project (i.e., 30 early-stage and 11 late-stage). Most ECRs were employed as researchers from research institutes (n=17), PhDs (n=12), or Post-Docs (n=6) or tenure track positions at Universities (n=6) who had a primary role to contribute in the scientific work packages of MYRIAD-EU to develop and test new methods and tools or other deliverables and milestones. Some of the late-stage ECRs have been involved in the proposal writing for MYRIAD-EU and took on management responsibilities for specific work packages or the project as a whole.  2.1. The process of collecting learnings and reflections

 In the case study we draw on perspectives and experiences of ECRs at various career stages, as well as those of senior researchers. These insights were collected on multiple occasions and in various formats over the past four years, including informal conversations among ECRs and reflection sessions at various meetings (e.g., general assemblies) within the aforementioned project context. Additionally, we shared and validated our ideas on examples and key factors in a broader context through a short course at the European Geosciences Union (EGU) General Assembly (EGU25; Vienna, Austria) in April 2025. Since the beginning of MYRIAD-EU, an active network has been established among the ECRs. Prior to each project general assembly, they organized a dinner together to connect, share experiences, and discuss project progress, their work, amongst other things. Additionally, they held occasional online check-in talks, roughly once or twice a year, with the explicit purpose of sharing recent successes or challenges, providing support, and brainstorming ideas for upcoming project activities. These meetups established trust among ECRs and provided the ability to share experiences as an ECR navigating their career path. Although no formal notes had been taken during these dinners or informal meetings, they collectively shaped a picture of the state of ECRs in the project.  Additionally, the ECRs organized a 2-hour workshop in the third year of the project. This workshop provided an opportunity to collect reflections on past experiences as ECRs in the project. They noted aspects they appreciated most, reflections they had regarding their role in the project, and aspects they thought would like to see changed, whether by themselves or by others in the project. The workshop also included a reflection on the remaining time in the project, where ECRs could share their plans, hopes, and wishes regarding their roles in the project, things to learn or to be involved in.  To validate key examples identified by the ECRs themselves in their own workshop, a session at the final project general assembly was organized which engaged with the entire project consortium to reflect on the involvement of ECRs in the project. The session aimed to collect instances where ECR involvement was noticed and which positive or unexpected experiences were made. Project partners were also encouraged to suggest ideas for future empowerment. During this session, project partners had the opportunity to comment on and reflect upon the notes contributed by others.  Additionally, they were tasked with ranking the collected aspects in terms of their perceived importance, ensuring these insights aligned with the broader consortium perspectives. 2.2. Positionality

 Our background and experiences shape the knowledge we produce as researchers (see e.g., Hausermann & Adomako, 2022). We are a team of co-authors working in disaster risk research, with disciplinary foundations in engineering or the natural sciences and varying experience in qualitative and quantitative methodologies. All authors were directly involved in the implementation of the MYRIAD-EU project and associated activities. We therefore write from embedded positions: as ECRs ourselves, as project managers or advisors responsible for meeting the project’s ambitions, and as members of an ECR board tasked with representing ECR interests in the management processes. Many of us are or were affiliated with the coordinating institution, which may shape how we interpret and present the project’s outcomes.  The study did not follow a predefined qualitative research design, nor was it conducted by an external team independent of the project’s internal hierarchies and dependencies. As such, academic power dynamics, institutional affiliations, professional relationships and the disciplinary context may have influenced both how experiences were expressed and how they are interpreted in this study. During the writing process, individual accounts were compared and discussed among co-authors in an effort to surface differing perspectives and critically question our interpretations. Nevertheless, the conclusions presented remain shaped by our shared disciplinary background, professional interdependencies, and collective investment in the project’s success. As a result, we focus on promising empowerment examples rather than questioning what could have gone better. This might already be a very important learning and insight for new projects to take into account.  —

 RC2.3: 

 Another flaw is the fact that the core themes in the topic are not strongly underpinned by empirical or theoretical work. The nature of evidence presented is quite ambiguous. For instance, although the paper states that insights were gleaned from reflections, etc., it does not clarify how these reflections were gathered and analysed. 

**AR2.3**: As part of the format adjustment, the added section is more explicit on the “data collection” process as discussed and presented in AR1.3 (see TR2.2).  —

 RC2.4: 

 Finally, this does not read more like an open letter as indicated earlier. It is more of a reflective case study.

**AR2.4**: We agree and thus changed the article format, see also our elaboration on that matter in **AR2.3** —

 RC2.5: 

 Certain key areas could be improved to better position this manuscript to contribute to the body of knowledge. 

**AR2.5**: We thank the reviewer for the feedback. As noted in the prior comments, we have addressed the key areas identified to strengthen the manuscript’s contribution to the body of knowledge. In particular, we have revised the text to more clearly position our work within the broader literature, with particular attention to the points raised in earlier and comments to other reviewers too (e.g., RC1.5, RC1.7, and RC2.3). —

 RC2.6: 

 Please consider strengthening its alignment with the format for an open letter. 

**AR2.6**: Instead of strengthening the alignment with an open letter format, we adjusted the format to one for a case study article as argued and elaborated upon in AR2.3. —

 RC2.7: 

 The core themes (e.g., ECR Boards, Agency, Mentorship, Networking, etc.) could be well-situated within the current state-of-the-art. 

**AR2.7**: We thank the reviewer for their comment. We have enhanced the discussion of the identified themes and key factors when relevant literature is available. Given the absence of existing literature on examples similar to those employed in the scope of MYRIAD-EU, we want to emphasise the novelty of our work in filling this gap. Our findings, reflections, and recommendations do align with broader literature, particularly aligning with a recent project which aimed at driving transformative change across the European research landscape. We've further highlighted and added context to this in “5.2 Ideas on how funding bodies could incentivize empowerment within projects” (see TR2.7).

 TR2.7:

 5. Looking Ahead

 “[...] 

 Our recommendations are in line with those proposed by Pizzolato et al. (2023) as part of the SOPs4RI (Standard Operating Procedures for Research Integrity) project, which aimed to drive transformative change across European Research Performing Organisations and Research Funding Organisations . Specifically, we suggest that the European Commission and other funding agencies should: (1) establish support systems for the skill development and capacity building of ECRs, (2) facilitate the involvement of ECRs in decision-making roles, and (3) provide bridge funding to support researchers during fixed-term contracts. This would enable ECRs to participate in encouragement and engagement activities, such as those outlined in our MYRIAD-EU case study.

 [...]

 ”

 —

 RC2.8: 

 The nature and scope of the reflections could be further clarified.

**AR2.8:** As part of the new section outlining the case study and data collection process, we also add a section on our positionality highlighting potential biases in the analysis, perception and discussion of aspects (see AR2.3). Additionally, we refined the purpose of the study to more explicitly outline the nature and scope of the reflections.

 —

# Reviewer report: Reviewer response for version 1

**DOI:** 10.21956/openreseurope.23275.r62364

**Published:** 2025-11-13

**Authors:** Leanne Gibbs

**Affiliations:** 1 School of Education, Charles Sturt University, Bathurst, New South Wales, Australia

Thank you for the opportunity to review this Open Letter. Writing on ECRs and their experience is very valuable in the current climate where the experience of ECRs is critical to workforce sustainability in Universities. 

 I have checked the guidelines for submission and do not feel it is entirely compliant.

*The Open Letter Open letters are short, peer-reviewed articles discussing policies relevant to a broad research community, presenting guidelines or white papers, or announcing new initiatives.*

 I believe the letter is successful in presenting guidelines or strategies but these represented an application of strategies in one project. It would be useful to present a contextual view here with some detail on the nature of the ECRs and the work they were required to engage with. This would offer, as mentioned, a more fine grained view and the possibility of application in alternative contexts.

*An Open Letter should usually represent the views of an eligible project or group of projects; publication does not imply endorsement by the European Commission.*

*An Open Letter should not include new data and analysis (if it does, a Research Article may be more suitable). The decision whether an Open Letter meets these criteria and is suitable for publication and subsequent peer review ultimately lies with F1000’s Head of Publishing*.

 This is an account of a project rather than an account of the views of the group. I think the Open Letter would benefit from the presentation of views from the large team and how successful strategies were in their opinion. I was also interested in a view as to which strategies were useful for the different components of the ECR work.

 Further citation and links to existing scholarship would be valuable. Although the format is different to an article, without citations or some context, the work reads more like a report of what worked and what didn't.

 I believe it would also be valuable to discuss time and the possibility of exploitation of ECRs. Whilst agency and empowerment are critical to ECR advancement, it is also important to consider these in the context of resources available to ECRs and the need to constrain the obligations on ECRs so that they are able to pursue their own research if this is different to the research of the project.

 There is also a silence here on sponsorship and it would be valuable to have some insight into how sponsorship plays a role.

 I appreciated the presentation of the themes and believe these could assist in a project with ECRs or in the supervision of ECRs. I would have found value in linking the themes to existing scholarship.

 Thank you for considering this feedback.

Where applicable, are recommendations and next steps explained clearly for others to follow? (Please consider whether others in the research community would be able to implement guidelines or recommendations and/or constructively engage in the debate)

Yes

Does the article adequately reference differing views and opinions?

Partly

Are all factual statements correct, and are statements and arguments made adequately supported by citations?

Partly

Is the rationale for the Open Letter provided in sufficient detail? (Please consider whether existing challenges in the field are outlined clearly and whether the purpose of the letter is explained)

Yes

Is the Open Letter written in accessible language? (Please consider whether all subject-specific terms, concepts and abbreviations are explained)

Yes

Reviewer Expertise:

Leadership in ECEC, ECRs, Research groups

I confirm that I have read this submission and believe that I have an appropriate level of expertise to confirm that it is of an acceptable scientific standard, however I have significant reservations, as outlined above.

# Response

**Published:** 2026-04-13

**Authors:** Kelley De Polt

Legend: RC -Reviewer Comment A - Author Response TR - Text Revision Report 1:  (Leanne Gibbs, Charles Sturt University, Victoria, New South Wales, Australia; link)   —

 RC1.1: Thank you for the opportunity to review this Open Letter. Writing on ECRs and their experience is very valuable in the current climate where the experience of ECRs is critical to workforce sustainability in Universities.    AR1.1: We thank the reviewer for their thoughtful engagement with our work, as well as their appraisal of the value of this topic in the current academic landscape. We have addressed the comments of all reviewers as outlined in our responses. Additionally, we improved the readability and clarity of the text by some minor textual adjustments throughout the manuscript. The main modifications are as follows:

- We changed the format of the study to a case study framing to better align with the journal guidelines and better capture the scope and narrative of the manuscript.
- We added many additional references throughout the manuscript to better place our findings in the broader context of the scholarship. 
- We added critical reflections on the insights produced, notably by adding a positionality statement.

In the following, we offer point-by-point responses. Responses to the review are written in a red font and numbered as AR (author response). Modifications in the text (TR, textual response) are marked in a blue font. In cases where smaller modifications within an existing paragraph are made, the changes are underlined; in cases where a new paragraph or section is added, we refrained from underlining for the sake of readability.  

 —RC1.2:  I have checked the guidelines for submission and do not feel it is entirely compliant. The Open Letter Open letters are short, peer-reviewed articles discussing policies relevant to a broad research community, presenting guidelines or white papers, or announcing new initiatives.   AR1.2: We thank the reviewer for this comment. Upon closer reflection of the different article formats, we agree that the chosen format is not the best suited one. As we will elaborate in more detail in respect to other reviewer comments (RC1.4; RC2.4; RC2.6), we decided to adjust the format to match the purpose and outline of a case study paper. The major adjustment in response is thus the addition of a new section (2. The case of MYRIAD-EU) which is a more explicit introduction of the case study and the data collection approach.  

 — RC1.3: I believe the letter is successful in presenting guidelines or strategies but these represented an application of strategies in one project. It would be useful to present a contextual view here with some detail on the nature of the ECRs and the work they were required to engage with. This would offer, as mentioned, a more fine grained view and the possibility of application in alternative contexts.   AR1.3: We have added a new section, “2. The Case of MYRIAD-EU” (TR1.3) which includes subsections “2.1. The Process of Collecting Learnings and Reflections” and “2.2. Positionality”. In Section 2, we elaborate on the roles of ECRs within the project. Specifically, we detail that many ECRs were engaged in PhD research directly related to MYRIAD-EU, while mid-to-late stage ECRs took on responsibilities such as project or work package management. Additionally, other ECRs contributed as general researchers, focusing on specific tasks and milestones. This elaboration aims to provide a clearer understanding of the diverse roles ECRs played, enhancing the applicability of our strategies to other contexts.  

 TR1.3: 2. The case of MYRIAD-EU MYRIAD-EU (Ward et al., 2022) was a four-year, EU Horizon 2020-funded project with a consortium composed of 19 project partners from universities, research institutions, NGO, and the private sector. The project aimed to provide policymakers, decision-makers, and practitioners with practical tools to create forward-looking disaster (multi-)risk management strategies. Central to the project were pilot study teams that tested methods developed within the project through a collaborative, co-design process with local stakeholders, addressing region- and sector-specific sustainability challenges. In order to maximize the impact of the project, one of the measures implemented was to ensure a “prominent role for Early Career Researchers within the management structure” as promised in the project proposal. Consequently, the representation of ECRs and the explicit consideration of their interests, ideas and needs were viewed as a critical component of the project’s success. As a result, the project offers a promising step towards effective ECR empowerment that benefits both the ECRs and the project, making it a valuable and relevant case to study and reflect upon. Altogether, the project involved 133 participants, of which about 30% were considered ECRs at the beginning of the project (i.e., 30 early-stage and 11 late-stage). Most ECRs were employed as researchers from research institutes (n=17), PhDs (n=12), or Post-Docs (n=6) or tenure track positions at Universities (n=6) who had a primary role to contribute in the scientific work packages of MYRIAD-EU to develop and test new methods and tools or other deliverables and milestones. Some of the late-stage ECRs have been involved in the proposal writing for MYRIAD-EU and took on management responsibilities for specific work packages or the project as a whole.    2.1. The process of collecting learnings and reflections In the case study we draw on perspectives and experiences of ECRs at various career stages, as well as those of senior researchers. These insights were collected on multiple occasions and in various formats over the past four years, including informal conversations among ECRs and reflection sessions at various meetings (e.g., general assemblies) within the aforementioned project context. Additionally, we shared and validated our ideas on examples and key factors in a broader context through a short course at the European Geosciences Union (EGU) General Assembly (EGU25; Vienna, Austria) in April 2025.   Since the beginning of MYRIAD-EU, an active network has been established among the ECRs. Prior to each project general assembly, they organized a dinner together to connect, share experiences, and discuss project progress, their work, amongst other things. Additionally, they held occasional online check-in talks, roughly once or twice a year, with the explicit purpose of sharing recent successes or challenges, providing support, and brainstorming ideas for upcoming project activities. These meetups established trust among ECRs and provided the ability to share experiences as an ECR navigating their career path. Although no formal notes had been taken during these dinners or informal meetings, they collectively shaped a picture of the state of ECRs in the project.    Additionally, the ECRs organized a 2-hour workshop in the third year of the project. This workshop provided an opportunity to collect reflections on past experiences as ECRs in the project. They noted aspects they appreciated most, reflections they had regarding their role in the project, and aspects they thought would like to see changed, whether by themselves or by others in the project. The workshop also included a reflection on the remaining time in the project, where ECRs could share their plans, hopes, and wishes regarding their roles in the project, things to learn or to be involved in.    To validate key examples identified by the ECRs themselves in their own workshop, a session at the final project general assembly was organized which engaged with the entire project consortium to reflect on the involvement of ECRs in the project. The session aimed to collect instances where ECR involvement was noticed and which positive or unexpected experiences were made. Project partners were also encouraged to suggest ideas for future empowerment. During this session, project partners had the opportunity to comment on and reflect upon the notes contributed by others.  Additionally, they were tasked with ranking the collected aspects in terms of their perceived importance, ensuring these insights aligned with the broader consortium perspectives.   2.2. Positionality Our background and experiences shape the knowledge we produce as researchers (see e.g., Hausermann & Adomako, 2022). We are a team of co-authors working in disaster risk research, with disciplinary foundations in engineering or the natural sciences and varying experience in qualitative and quantitative methodologies. All authors were directly involved in the implementation of the MYRIAD-EU project and associated activities. We therefore write from embedded positions: as ECRs ourselves, as project managers or advisors responsible for meeting the project’s ambitions, and as members of an ECR board tasked with representing ECR interests in the management processes. Many of us are or were affiliated with the coordinating institution, which may shape how we interpret and present the project’s outcomes.    The study did not follow a predefined qualitative research design, nor was it conducted by an external team independent of the project’s internal hierarchies and dependencies. As such, academic power dynamics, institutional affiliations, professional relationships and the disciplinary context may have influenced both how experiences were expressed and how they are interpreted in this study. During the writing process, individual accounts were compared and discussed among co-authors in an effort to surface differing perspectives and critically question our interpretations. Nevertheless, the conclusions presented remain shaped by our shared disciplinary background, professional interdependencies, and collective investment in the project’s success. As a result, we focus on promising empowerment examples rather than questioning what could have gone better. This might already be a very important learning and insight for new projects to take into account.   

 — RC1.4:  An Open Letter should usually represent the views of an eligible project or group of projects; publication does not imply endorsement by the European Commission. An Open Letter should not include new data and analysis (if it does, a Research Article may be more suitable). The decision whether an Open Letter meets these criteria and is suitable for publication and subsequent peer review ultimately lies with F1000’s Head of Publishing.   This is an account of a project rather than an account of the views of the group. I think the Open Letter would benefit from the presentation of views from the large team and how successful strategies were in their opinion. I was also interested in a view as to which strategies were useful for the different components of the ECR work.   AR1.4: As indicated in response to AR1.2, we adjusted the article format to one of a case study. Open Research Europe describes case study publications as those which “[describe] actual interventions or experiences”. And they “accept case studies examining a person, place, event, phenomenon, or other type of subject of analysis in order to highlight key themes”. In our paper, we primarily present the case of MYRIAD-EU sharing reflections and lessons from the different ways ECR empowerment took place and how it benefitted both the ECRs and the project. As such a case study format seems to match the requirements by Open Research Europe. At the same time, our study is not a typical case study experiment which can easily be reproduced. It is rather a unique account of a specific, complex process and its outcomes which is why we originally opted for an Open research letter.    In order to adjust the format, we add a new section 2 (please see above TR1.3) outlining the case study, the method for data collection and the data collection events themselves along with a statement on the positionality of our findings.    As such we will make more explicit that the findings and reflections in this study are an account of views of the large project team, done via repeated informal check-in meetings between ECRs where they shared recent successes, challenges and general reflections regarding their views on the progress of the project, along with a dedicated workshop where ECRs reflected on their insights regarding ECR empowerment in the project and one workshop that had been conducted at the final project meeting with all project partners, where we together reflected on the key needs and benefits of ECR empowerment in the context of research projects.    We agree that a reflection on the successfulness of different strategies is an interesting aspect of the analysis and we believe we have done this, particularly when considering the suggestions we make for other projects interested in following the example of MYRIAD-EU. Similarly, we doubt it would be helpful to discuss the ‘success’ in more detail as the project did not set out a set of objectives regarding ECR empowerment or we did not measure success, e.g. by means of KPI’s. We added an additional paragraph as part of section 5 (Looking ahead) (TR1.4) explicitly touching upon the lack of systematic measurement of the success and performance gain due to efforts of ECR empowerment.   

 TR1.4: 5. Looking Ahead “[...]  Our recommendations are in line with those proposed by Pizzolato et al. (2023) as part of the SOPs4RI (Standard Operating Procedures for Research Integrity) project, which aimed to drive transformative change across European Research Performing Organisations and Research Funding Organisations . Specifically, we suggest that the European Commission and other funding agencies should: (1) establish support systems for the skill development and capacity building of ECRs, (2) facilitate the involvement of ECRs in decision-making roles, and (3) provide bridge funding to support researchers during fixed-term contracts. This would enable ECRs to participate in encouragement and engagement activities, such as those outlined in our MYRIAD-EU case study. [...] ”  

 — RC1.5: Further citation and links to existing scholarship would be valuable. Although the format is different to an article, without citations or some context, the work reads more like a report of what worked and what didn't.   AR1.5: We thank the reviewer for this feedback. Throughout the manuscript, especially in the introduction as well as in “4. Key factors that enabled empowerment within MYRIAD-EU” and beyond  which reflects on the underlying factors driving successful implementation, we’ve added a range of references to existing scholarship to contextualize but also further support the findings and suggestions we identified in the case of MYRIAD-EU. In other sections, we believe they are less needed given the adjusted format of the study, which is a case study work.    Additional references included:

- Addy, T. M., Addy, N. A., Hendrickson, B., & Mitchell, K. A. (2023). Supporting mentees through inclusive mentorship. New Directions for Teaching and Learning, 2023(175), 11-19. https://doi.org/10.1002/tl.20554
- Alderson, D., Clarke, L., Schillereff, D., & Shuttleworth, E. (2022). Navigating the academic ladder as an early career researcher in earth and environmental sciences. Earth Surface Processes and Landforms, 48(2), 475–486. https://doi.org/10.1002/esp.5497
- Atenas, J., Nerantzi, C., & Bussu, A. (2023). A conceptual approach to transform and enhance academic mentorship: Through open educational practices. Open Praxis, 15(4), 271-287. https://doi.org/10.55982/openpraxis.15.4.595
- Antes, A. L., Mart, A., & DuBois, J. M. (2016). Are leadership and management essential for good research? An interview study of genetic researchers. Journal of Empirical Research on Human Research Ethics, 11(5), 408-423. https://doi.org/10.1177/1556264616668775
- Bálint, E., Csuka, D., Venglovecz, V., Schlosser, G., Lázár, Z., Gselmann, E., Alpár D., & Solymosi, K. (2021). Six reasons to launch a Young Academy. Nature, 594, 599-601. https://doi.org/10.1038/d41586-021-01682-9
- Bozeman, B. & Corley, E. (2004). Scientists’ collaboration strategies: implications for scientific and technical human capital. Research Policy, 33(4), 599-616. https://doi.org/10.1016/j.respol.2004.01.008
- Chatzea, V. E., Mechili, E. A., Melidoniotis, E., Petrougaki, E., Nikiforidis, G., Argyriadis, A., & Sifaki-Pistolla, D. (2022). Recommendations for young researchers on how to better advance their scientific career: A systematic review. Population Medicine, 4(September), 1-17. https://doi.org/10.18332/popmed/152571
- Cvitanovic, C., Blythe, J., van Putten, I., Maddison, L., Bopp, L., Brodie, S., Fulton, B. A., Lopes, P. F., Pecl, G., Penca, J., & Sumaila, U. (2024). Building Successful International Summer Schools to Enhance the Capacity of Marine Early Career Researchers. Ocean and Society, 1, Article 9328. https://doi.org/10.17645/oas.9328
- Erdem, F., & Aytemur, J. Ö. (2008). Mentoring—A relationship based on trust: Qualitative research. Public Personnel Management, 37(1), 55-65. https://doi.org/10.1177/009102600803700104
- Fisher, J. J., & James, J. L. (2022). Know the game: insights to help early career researchers successfully navigate academia. Placenta, 125, 78-83.  https://doi.org/10.1016/j.placenta.2021.10.013
- Færgeman, N.J., Christiansen, T.S.B. & Repasky, G. (2025). Empowering early career researchers: academy-driven pathways to excellence. Nature Reviews Endocrinology, 21, 325–326. https://doi.org/10.1038/s41574-025-01110-2
- Hausermann, H., & Adomako, J. (2022). Positionality, ‘the field,’ and implications for knowledge production and research ethics in land change science. Journal of Land Use Science, 17(1), 211–225. https://doi.org/10.1080/1747423X.2021.2015000
- Martin, A., Mori, J., & Froehlich, D. E. (2023). Career development of early career researchers via distributed peer mentoring networks. Merits, 3(3), 569-582. https://doi.org/10.3390/merits3030034
- Parsons, C. & O’Conner, P. (2023) You’ve heard of mentorship in science, but what about sponsorship?. Nature. https://doi.org/10.1038/d41586-023-00123-z
- Pizzolato, D., Reyes Elizondo, A., Aubert Bonn, N., et al. (2023). Bridging the gap – how to walk the talk on supporting early career researchers. Open Research Europe, 3(75). https://doi.org/10.12688/openreseurope.15872.1
- Young, K. J. (2014). Research mentoring: Suggestions and encouragement from a reflection exercise. Journal of Chiropractic Education, 28(2), 168-172. https://doi.org/10.7899/JCE-14-7

  — RC1.6:  I believe it would also be valuable to discuss time and the possibility of exploitation of ECRs. Whilst agency and empowerment are critical to ECR advancement, it is also important to consider these in the context of resources available to ECRs and the need to constrain the obligations on ECRs so that they are able to pursue their own research if this is different to the research of the project. There is also a silence here on sponsorship and it would be valuable to have some insight into how sponsorship plays a role.   AR1.6: We appreciate the suggestion regarding additional discussion on key factors involved in the engagement and empowerment of ECRs. In response to the addition of literature on exploitation, we have added to our discussion in Section 4.3 “External factors in the project context” (TR1.6), by adding information on resource allocation, particularly focusing on funding and administrative support. This addition highlights how these factors can impact ECRs development and opportunities and the measures taken to prevent their exploitation. Prior literature has validated the link between exploitation to these elements. Regarding sponsorship, we have integrated this concept into Section 4.1 “Direct influences and advisory support” (TR1.6). We discuss the importance of trust between advisors and ECRs, emphasising how sponsorship requires the advisors to trust ECRs, to facilitate the leveraging of their resources and especially networks.   

 TR1.6: 4.1 Direct influences and advisory support  [...] Being an effective advisor also means being open and inclusive (Addy et al., 2023; Atenas et al., 2023) and considerate of the boundaries ECRs set - especially with regards to their interests and availability. Acknowledging that ECRs are a diverse group of individuals involved in the research process can help tailor strategies to different experiences, reduce the risk of exploitation and empower individuals, for example, by acknowledging (non-)science activities that contribute to professional development (Fischer et al., 2022; Antes et al., 2016). [...]   4.3 External factors in the project context [...] Another essential factor is the availability of resources. Funding is crucial in determining one’s capacity for participation, mobility, and available time to activities beyond the core responsibilities in their role. Especially in the current academic climate, where the “publish-or-perish” culture persists, ECRs are particularly vulnerable to exploitation due to the pressures to publish to advance (their) careers and to quantify their accomplishments. This is exacerbated given concerns around short-term contracts, work-life balance, and existing inequalities (Alderson et al., 2023). Therefore, it is crucial to have appropriate and flexible funding strategies in place, and accompanied by appropriate levels of administrative support. This ensures that essential resources are accessible and can be utilised to provide opportunities for ECRs as they progress through a project.   

 — RC1.7:  I appreciated the presentation of the themes and believe these could assist in a project with ECRs or in the supervision of ECRs. I would have found value in linking the themes to existing scholarship.   AR1.7: We thank the reviewer for their feedback. We appreciate the suggestion to link the themes more explicitly to existing scholarship. In adjusting the format of the manuscript from an open letter to a case study (as suggested by comments RC1.2; RC1.4), the key themes we identified are to recount the MYRIAD-EU experiences. In response, we have integrated additional literature into the discussion of the synthesised key factors and recommendations for future projects in looking ahead (see also AR1.5). We believe this aligns with the suggestion and adds to the manuscripts place setting in the literature.  

 — RC1.8:  Thank you for considering this feedback.   AR1.8: We thank the reviewer for their feedback and have taken all of it into consideration. —
